# Depression symptom profiles and associated sociodemographic factors among perinatal women: A latent profile analysis

**DOI:** 10.1016/j.aprim.2026.103550

**Published:** 2026-06-15

**Authors:** Chao Xue, Jiashu Liu, Hua Shao, Shulan Tu

**Affiliations:** a School of Educational Science, Quanzhou Normal University, Quanzhou, Fujian, China; b Psychological Development Guidance Center, Quanzhou Normal University, Quanzhou, Fujian, China

Perinatal depression (PND) is one of the most common mental health problems during pregnancy and the postpartum period and represents an important public health concern.^1^ Previous studies have shown that depressive symptoms during the perinatal period are heterogeneous, ranging from mild emotional distress to clinically significant depressive symptoms.^2^ Sociodemographic factors such as age, educational attainment, and marital status may influence vulnerability to perinatal depression.^3^ However, large-sample studies examining depressive symptom profiles and their associated demographic characteristics remain limited. Therefore, this study aimed to identify latent profiles of depressive symptoms among perinatal women and examine their associated sociodemographic factors.

A cross-sectional survey was conducted among 10,652 perinatal women (from pregnancy to 2 months postpartum) receiving care in Fujian Province, China, between 2022 and 2023. Depressive symptoms were assessed using the Patient Health Questionnaire-9 (PHQ-9).^4^ Latent profile analysis (LPA) was performed based on the nine PHQ-9 items to identify depressive symptom subtypes. Multinomial logistic regression was subsequently used to examine associations between latent classes and demographic variables including age, educational attainment, and marital status.

Three latent profiles were identified: No Depression (81.87%, *n* = 8721), Mild Depression (15.27%, *n* = 1627), and Moderate Depression (2.85%, *n* = 304) (Fig. 1). The three-class model was selected as the optimal solution based on lower AIC, BIC, and adjusted BIC values, significant LMR-LRT and BLRT results, high entropy (0.965), and model parsimony. Mean depression scores increased progressively across profiles (No Depression: 1.23 ± 1.69; Mild Depression: 9.49 ± 3.11; Moderate Depression: 12.78 ± 5.35; *F* = 13,463.89, *p* < .001). Compared with women older than 34 years, younger women were more likely to belong to the Mild Depression profile (<20 years: *OR* = 1.69, 95% CI 1.24–2.30; 20–34 years: *OR* = 1.41, 95% CI 1.19–1.64). Lower educational attainment (high school or below) was associated with increased likelihood of belonging to both the Mild Depression (*OR* = 1.45, 95% CI 1.30–1.62) and Moderate Depression (*OR* = 1.60, 95% CI 1.26–2.02) profiles. Married women showed lower likelihood of belonging to the Mild Depression profile (*OR* = 0.36, 95% CI 0.22–0.57). Educational attainment demonstrated the strongest and most consistent association with depressive symptom profiles.

**Figure 1:**
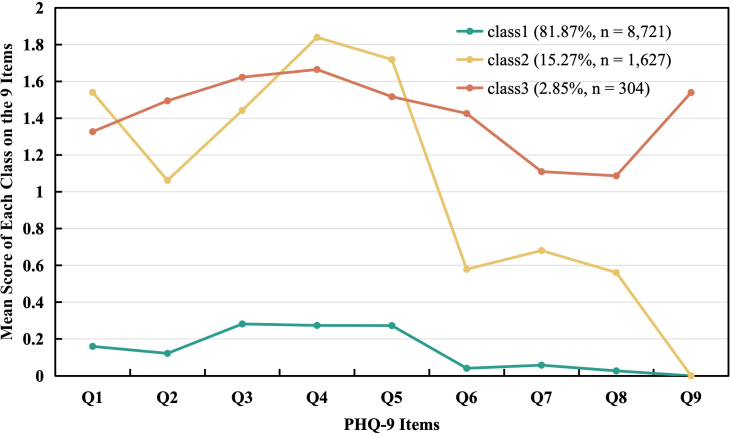
Latent profiles based on the PHQ-9 in a perinatal sample (*n* = 10,652). The line plot depicts the mean scores (*y*-axis) on each of the nine PHQ-9 items (*x*-axis) for the three identified classes. The estimated class prevalences were as follows: Class 1 (No Depression, 81.87%), Class 2 (Mild Depression, 15.27%), and Class 3 (Moderate Depression, 2.85%).

This study identified substantial heterogeneity in depressive symptoms among perinatal women. Although most participants reported minimal depressive symptoms, approximately one in six women experienced mild depressive symptoms, while a smaller subgroup exhibited moderate depressive symptoms requiring greater clinical attention. The main finding was that lower educational attainment showed the strongest and most consistent association with depressive symptom profiles.

This association may be explained by differences in access to health information, coping strategies, and psychosocial resources. Women with lower educational attainment may have fewer cognitive and social resources to cope with pregnancy-related stressors, thereby increasing vulnerability to depressive symptoms. Younger women may be at greater risk of depressive symptoms during pregnancy and early motherhood, whereas marital support may buffer psychological distress during the perinatal period.^5^

The cross-sectional design does not allow causal interpretation of the associations between demographic factors and depressive symptoms. In addition, the study did not assess variables such as parity, household income, or marital satisfaction, which may influence perinatal mental health outcomes. Further longitudinal studies are needed to investigate the trajectories of depressive symptom profiles across pregnancy and postpartum stages.

These findings highlight the unmet need for early screening and targeted psychosocial interventions for younger, less educated, and socially vulnerable perinatal women. Personalized prevention and intervention strategies may help improve maternal mental health outcomes during the perinatal period.^6^

## Ethics approval and consent to participate

The study complies with the Declaration of Helsinki and has approved by the Ethics Committee of Quanzhou Normal University (QZSYLL202215), and all participants provided online informed consent prior to participation.

## Generative AI disclosure

No generative AI was used in the preparation of this manuscript.

## Funding

10.13039/501100002827Fujian Province College Students’ Innovation and Entrepreneurship Training Program (S202510399040); Quanzhou Social Science Planning Project (Grant No. 2025E14); Major Project of the Marxist Theory Research and Construction Project, Fujian Provincial Social Science Foundation (FJ2024MGCA044).

## Conflict of interests

The authors declare no competing interests.
